# Objective response rates, while useful, may not fully reflect the value of psoriasis treatments

**DOI:** 10.1016/j.jdin.2026.03.012

**Published:** 2026-03-28

**Authors:** Mahak Sharma, Noah Z.H. Feldman, Steven R. Feldman

**Affiliations:** a New York Institute of Technology College of Osteopathic Medicine, Jonesboro, Arkansas; b Department of Statistics, The Pennsylvania State University, State College, Pennsylvania; c Center for Dermatology Research, Department of Dermatology, Wake Forest University School of Medicine, Winston-Salem, North Carolina; d Department of Pathology, Wake Forest University School of Medicine, Winston-Salem, North Carolina; e Department of Public Health Sciences, Wake Forest University School of Medicine, Winston-Salem, North Carolina; f Department of Dermatology, University of Southern Denmark, Odense, Denmark

**Keywords:** biologics, DLQI, PASI, patient-reported outcomes, psoriasis, statistics

*To the Editor:* Psoriasis Severity and Area Index (PASI) is a standard metric to assess treatments for chronic plaque psoriasis. Recent clinical trials may define treatment success as achieving 90% improvement in PASI (PASI90), as the outcome is associated with greater improvement in quality of life (QoL) than achieved by patients reaching PASI scores 75 to 89.^1^ Dichotomous objective success measures are valuable and needed for intent-to-treat analyses. These measures can also provide for a standardized comparison between therapies; however, the objective measures may not always correlate directly with patients’ subjective perceptions of improvement. To assess the relative subjective improvement associated with treatments of different objective efficacy, we compared the physician-reported objective improvements in disease severity (PASI90) with the patient-reported subjective improvements in the Dermatology Life Quality Index (DLQI) across multiple medications.

Published data provided PASI90 and DLQI score outcomes at weeks 12 or 16 for chronic plaque psoriasis treatments.^2^, ^3^, ^4^, ^5^, ^6^, ^7^, ^8^, ^9^, ^10^, ^11^ Among the 9 treatments, PASI90 rates ranged broadly from 9% to 73%, while DLQI score improvements ranged more narrowly from 59% to 86% (Fig 1). Treatments with low PASI90 success rates still provided considerable DLQI score improvement. (Fig 2).

**Fig 1:**
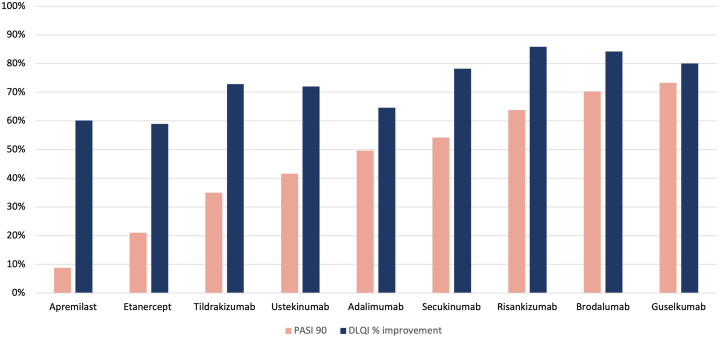
PASI90 and DLQI treatment outcomes for 9 psoriasis therapies. There is a wide range of PASI90 scores for the different treatments. The most effective treatment had PASI90 rates 8 times higher than the least effective treatment. However, there is far less difference in DLQI scores, with the most effective treatment offering about 25% more DLQI score improvement. *DLQI,* Dermatology life quality index; *PASI*, Psoriasis severity and area index. An analysis of variance (ANOVA) analysis was performed using both ordinary least squares and weighted least squares, which included sample sizes. Statistically significant results were obtained (*P*= .00223 for ordinary least squares [OLS]; *P*= .00323 for weighted least squares [WLS]), providing strong evidence against the null hypothesis. This suggests that the observed relationship is unlikely due to random variation alone. Usual model assumptions, including homoscedasticity, were satisfied. Diagnostics revealed several influential drugs, most notably risankizumab, which is expected, given the small sample size (*n*= 9).

**Fig 2:**
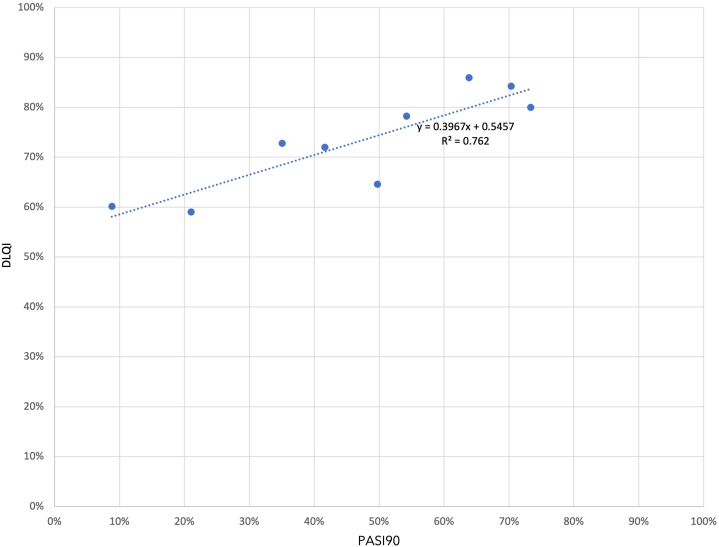
Association of PASI90 response rates with DLQI score improvement. The regression line slope implies that each additional 10% improvement in PASI90 yields about 4% improvement in quality of life. The *y-intercept* of 55% implies that drugs for which no one achieves PASI90 can still provide most of the improvement in quality of life. Even therapies that achieve a low rate of PASI90 success, such as apremilast (9%) and etanercept (21%), still offer considerable improvement in DLQI scores. *DLQI,* Dermatology Life Quality Index; *PASI*, Psoriasis Area and Severity Index.

A “high bar” objective improvement rate, like PASI90, is useful to differentiate the objective efficacy of highly effective treatments. However, there is diminishing subjective benefit with higher objective improvement, with modest objective improvement providing most of the QoL improvement of more effective treatments.

Improvements in DLQI score do not scale linearly with increased PASI90 achievement. Reliance purely on PASI90 success rates may lead to underestimating the benefits of older, and possibly more affordable, treatments. While PASI90 is associated with a statistically significantly greater percentage of patients with DLQI scores of 0 or 1 compared with patients who do not achieve PASI90, the absolute difference in DLQI score improvement may be small. Between the apremilast (PASI90: 9%) and guselkumab (PASI90: 73%) trials, the absolute DLQI score improvement difference is only 2.9, while the minimally clinically important difference in DLQI score is in the range of 3 to 5.^12^ In both trials, the DLQI score would define the average patient’s disease after treatment as having a “small impact on patient’s life.”

While more improvement in QoL is valuable, comparing therapies by PASI 90 or PASI100 outcomes alone may underestimate the value of alternatives—especially those with lower levels of PASI 90 achievement—in their ability to improve patients’ QoL.

## Conflicts of interest

Dr SR Feldman has received research, speaking and/or consulting support from Eli Lilly and Company, GlaxoSmithKline/Stiefel, AbbVie, Janssen, Alovtech, vTv Therapeutics, Bristol-Myers Squibb, Samsung, Pfizer, Alumis, Boehringer Ingelheim, Oruka, Amgen, Dermavant, Arcutis, Novartis, UCB, Helsinn, Sun Pharma, Almirall, Galderma, Leo Pharma, Mylan, Celgene, Ortho Dermatology, Menlo, Merck & Co, Qurient, Forte, Arena, Biocon, Accordant, Argenx, Sanofi, Regeneron, the National Biological Corporation, Caremark, Teladoc, BMS, Ono, Micreos, Eurofins, Informa, UpToDate, Verrica, and the National Psoriasis Foundation. He is the founder and part-owner of Causa Research and holds stock in Sensal Health. Drs Sharma and NZH Feldman have no conflicts to declare.
